# Topical Probiotics in Diabetic Wound Healing: Emerging Therapeutic Strategies

**DOI:** 10.3390/ijms27062826

**Published:** 2026-03-20

**Authors:** Eni Çelo, Aida Dama, Sokol Hasho, Leonard Deda

**Affiliations:** 1 Department of Internal Medicine, University Hospital of Trauma, 1000 Tirana, Albania; eni.celo89@gmail.com; 2 Department of Biomedical and Experimental Sciences, Faculty of Medicine, University of Medicine, 1005 Tirana, Albania; leonarddeda@hotmail.com; 3 Department of Pharmacy, Faculty of Medical Sciences, Albanian University, 1017 Tirana, Albania; 4 Department of Surgery, Shefqet Ndroqi University Hospital, 1030 Tirana, Albania; sokolphasho68@gmail.com

**Keywords:** diabetic foot ulcers, topical probiotics, wound healing, skin microbiome, biofilms, inflammation, angiogenesis

## Abstract

Diabetic foot ulcers (DFUs) are among the most serious and costly complications of diabetes, characterised by delayed healing, frequent infections, and a high risk of recurrence. Despite advances in wound care, many current therapies fail to address the multifactorial pathophysiology of diabetic wounds, including vascular dysfunction, immune dysregulation, chronic inflammation, and microbial imbalance. In this context, topical probiotics have emerged as a promising microbiome-based strategy aimed at restoring microbial balance while promoting tissue repair. This review summarises current evidence on the use of topical probiotics in diabetic wound healing, with a particular focus on DFUs, outlining key pathophysiological barriers to healing and examining how probiotic therapies may counteract these processes through antimicrobial, antibiofilm, immunomodulatory, and pro-angiogenic mechanisms. Preclinical studies suggest that topical probiotics may promote accelerated wound closure, reduce bacterial burden, modulate inflammatory responses, and enhance collagen deposition and angiogenesis following topical probiotic application. Early clinical studies investigations remain limited to small pilot studies and case series but have reported preliminary signals of enhanced healing and acceptable short-term tolerability in small exploratory cohorts. In addition, recent advances in probiotic delivery, such as bioengineered dressings, postbiotic formulations, and nano-enabled systems designed to improve stability and therapeutic performance, are also discussed. While existing data indicate biological plausibility and early clinical feasibility, larger, well-designed randomized controlled trials and deeper mechanistic investigations are still required to confirm efficacy, clarify safety in high-risk populations, and enable responsible clinical translation.

## 1. Introduction

Diabetes mellitus is a growing global health challenge, currently affecting more than 500 million adults, with numbers expected to rise sharply in the coming decades. Among its many complications, diabetic foot ulcers (DFUs) remain among the most disabling and costly. Around one in four people with diabetes will develop a foot ulcer during their lifetime [1], and many of these wounds fail to heal properly. This often leads to persistent infection, repeated recurrence, and in severe cases, lower-limb amputation. Beyond the physical consequences, diabetic wounds also carry heavy psychological, social, and economic costs which further highlight the urgent need for more effective treatment strategies.

Despite advances in wound care, the clinical management of diabetic wounds remains challenging. Standard management of diabetic wounds includes pressure offloading, surgical debridement, infection control, revascularization procedures, and advanced dressings [2]. However, even with appropriate care, healing is frequently delayed, and ulcer recurrence remains common. This reflects the complex pathophysiology of diabetic wounds which includes cellular dysfunction, altered inflammatory responses, oxidative stress, the formation of advanced glycation end-products and neurovascular abnormalities [3]. In addition, the development of antimicrobial resistance, which affects both clinical and therapeutic outcomes, with consequences ranging from treatment failures and the need for expensive and safer alternative drugs to the cost of higher rates of morbidity and mortality, longer hospitalization, and high-healthcare costs [4]. Together, these challenges underscore the critical need for new, locally acting therapies capable of addressing both the microbial and host components of impaired wound repair.

Chronic hyperglycemia is known to disrupt local immune function and alter the wound microbiome, contributing to persistent inflammation and delayed healing. This provides a biological rationale for exploring microbiome-targeted strategies aimed at restoring local microbial balance and supporting tissue repair [1,5,6]

Probiotics, defined by the World Health Organization as “live microorganisms which, when administered in adequate amounts, confer a health benefit on the host,” have traditionally been studied mainly in gut health. However, recent advances have expanded their application to dermatology and wound management, particularly topical formulations delivering beneficial microbes directly to the wound bed.

While classical *Lactiplantibacillus* species remain widely studied, recent research has broadened the probiotic repertoire to include commercially available strains such as *Bacillus clausii* (Enterogermina^®^), *Bacillus coagulans* (formerly marketed as “*Lactobacillus sporogenes*”) (Sporlac^®^), and *Bifidobacterium longum* (Florachamp^®^) formulated in biocompatible polyethylene glycol (PEG)–glycerol gels. These novel strains have demonstrated potent antimicrobial effects against common wound pathogens including *Staphylococcus aureus* and *Pseudomonas aeruginosa*, alongside significant enhancement of wound contraction, collagen deposition, and re-epithelialization in preclinical diabetic wound models, achieving healing efficacy comparable to standard treatments [7].

Topical probiotic therapies exhibit diverse biological activities: competition with pathogenic microbes for adhesion sites and nutrients, secretion of antimicrobial peptides and organic acids, modulation of immune responses to attenuate excessive inflammation, and stimulation of angiogenesis and tissue regeneration. Importantly, probiotic gels and dressings act locally at the wound surface, reducing systemic exposure and associated risks in vulnerable diabetic patients [8].

Nevertheless, given that many patients with DFUs present with impaired immunity, vascular compromise, and multiple comorbidities, careful consideration of safety and patient selection is essential, and probiotic-based approaches should currently be regarded as experimental adjuncts rather than established therapies, pending confirmation in larger controlled studies [2,8].

The aim of this review is to critically evaluate the emerging role of topical probiotics in diabetic wound healing, with particular attention to their application in DFUs. We summarize the key pathophysiological barriers to healing in diabetic wounds, review the available preclinical and clinical evidence on topical probiotic therapies, and discuss recent innovations, current limitations, and future directions for their translation into routine clinical practice.

## 2. Methods

This review was conducted as a narrative synthesis of available preclinical and clinical literature investigating topical probiotic and postbiotic approaches in diabetic wound healing. Rather than following a formal systematic review or meta-analysis design, this work was developed as a structured narrative synthesis designed to integrate mechanistic insights with preclinical and early clinical observations. A literature search was performed using PubMed, Scopus, and Web of Science databases, covering publications from January 2000 to January 2025. Search terms included combinations of “diabetic foot ulcer,” “topical probiotics,” “skin microbiome,” “postbiotics,” and “wound healing.”

The review included experimental animal studies, clinical trials, pilot studies, and case reports evaluating microbiome-based topical therapies in diabetic or chronic wounds. Studies focusing exclusively on systemic probiotic administration without topical application were excluded. Review articles were also consulted to help explain the underlying biological mechanisms. Only studies published in English were considered.

Given the limited number of randomized controlled trials and the emerging nature of this field, a narrative approach was adopted to integrate mechanistic insights with preclinical and early clinical data. Particular emphasis was placed on biological plausibility, reported outcomes, methodological limitations, and safety considerations. Due to the heterogeneity and early-stage preliminary nature of the available evidence, no formal quality scoring was applied, and conclusions were drawn cautiously.

Bacterial nomenclature throughout this manuscript follows the updated reclassification of the genus *Lactobacillus* proposed by Zheng et al. (2020) [9], with former species names retained in parentheses at first mention for clarity.

## 3. Pathophysiology of the Diabetic Wounds

Diabetic wounds develop due to a complex interplay of metabolic, vascular, neuropathic, and immune factors. These interacting mechanisms disrupt the normal sequence of tissue repair and lock the wound into a chronic non-healing state. Clinically, DFUs encompass distinct phenotypes, including neuropathic, ischemic, and neuro-ischemic wounds, each characterized by different degrees of vascular compromise, inflammation, and tissue hypoxia, which may influence microbial colonization patterns and responsiveness to microbiome-based interventions [2].

Microvascular and macrovascular damage play a leading role due to impaired tissue metabolism in diabetes mellitus (DM) [10]. Persistent hyperglycaemia induces endothelial dysfunction through activation of the polyol, hexosamine, advanced glycation end-products (AGEs), and protein kinase C (PKC) pathway [11]. Activation of these enzymes results in dysfunction of microarterioles that regulate the contractility of the smooth muscle of vessels supplying distal tissue areas [12]. This can be associated with delayed wound healing due to a restriction of oxygen and nutrient supply by impaired arteriole relaxation [13].

Hyperglycaemia-driven oxidative stress, impaired perfusion, altered wound pH, and diabetes-associated immune dysfunction collectively reshape the local wound microenvironment, creating conditions that favor opportunistic pathogens over protective commensal species. Reduced oxygen tension and nutrient imbalance further promote biofilm formation and microbial persistence, while defective innate immune responses limit effective bacterial clearance. Together, these factors establish a self-perpetuating cycle of dysbiosis and chronic inflammation that delays tissue repair and sustains wound chronicity [14,15,16].

Oxidative stress represents a key downstream consequence of these activated biochemical pathways. Signalling through the AGE/RAGE axis, polyol pathway, PKC activation, and the hexosamine pathway leads to excessive production of inflammatory mediators and profound structural changes in the microvasculature. These include pericyte degeneration, basement membrane thickening, endothelial hyperplasia, nitric oxide reduction, impaired vasodilation, and increased levels of procoagulant biomarkers such as IL-6, TNF-α, D-dimer, and PAI-1 [17]. Collectively, these alterations drive the development of diabetic microangiopathy. Structural damage at the capillary and arteriolar level further compromises the delivery of oxygen, nutrients, and activated immune cells to the tissues, increasing susceptibility to infection and accelerating both the onset and progression of diabetic ulcers.

In parallel, diabetic neuropathy significantly accelerates both ulcer formation and chronicity. The loss of protective sensation due to neuropathy and diminished trophic effect by neuropeptide deficiency lead to trauma and increased pressure on the foot skin and a diminished hyperemic response to tissue injury [18]. These alterations may cause acute wounds to advance to chronic wounds with impaired healing [18]. More recently, small fiber dysfunction has been shown to be an early feature in patients with type 2 diabetes and has also been implicated in delayed wound healing [19].

Under physiological conditions, wound healing is a finely coordinated process that works to control infection, clear away damaged tissue [20] and restores homeostasis and tissue function through four phases: haemostasis, inflammation, proliferation and remodelling [21,22]. In diabetes, this orderly sequence becomes profoundly disrupted. Recruitment and proliferation of progenitor cells are impaired, growth factor release is reduced, and new vessel formation is diminished [20,23]. Macrophages, which are key regulators of tissue repair, show delay in the production of chemokines and cytokines such as MCP-1 and MIP-2, resulting in delayed monocyte influx into the wound [24]. This delay leads to defective efferocytosis [25], allowing the accumulation of wound debris, apoptotic cells, and neutrophils. The prevalence of wound debris sets up a constant inflammatory phase which degrades the wound microenvironment [25]. Inflammation persists into the remodeling stage, preventing complete resolution of wound healing [24].

Failure of macrophages to transition from the pro-inflammatory M1 phenotype toward the reparative M2 phenotype is increasingly recognized as a central driver of diabetic wound chronicity. Persistent predominance of M1 macrophages sustains inflammatory cytokine signaling and tissue injury, while insufficient M2 polarization limits angiogenesis, extracellular matrix remodeling, and regenerative repair. Mechanistically, impaired macrophage plasticity in diabetic wounds has been linked to altered metabolic signaling and epigenetic regulation, reinforcing prolonged inflammatory states and delaying progression to proliferative healing phases [14,26].

In addition, neutrophil migration is slowed and macrophages efficiency is reduced, weakening host defense and increasing the vulnerability to infection. Additionally, advanced glycation end-products (AGEs) alter collagen structure, reducing tissue elasticity and repair capacity. Prolonged inflammation, impaired angiogenesis, and poor extracellular matrix remodeling contribute to the chronicity of diabetic wounds [22].

In addition to immune and vascular dysfunction, diabetic wounds exhibit features of premature cellular senescence, particularly affecting fibroblasts and keratinocytes, which limit proliferative capacity and delays tissue regeneration [27]. Endothelial progenitor cell (EPC) mobilization and homing to wound sites are impaired in diabetes, contributing to defective neovascularization and delayed granulation tissue formation [28]. Persistent AGE–RAGE signaling further amplifies oxidative stress and endothelial dysfunction while sustaining chronic inflammatory pathways and altering local tissue metabolism. Together these effects contribute to a wound environment that favors delayed repair and microbial persistence [29]. Moreover, diabetic wounds often display an alkaline shift in pH, which favors protease activity and pathogen persistence and may influence the stability and activity of applied microbiome-based therapies [30]. Together, cellular senescence, EPC dysfunction, AGE–RAGE-mediated vascular injury, and pH imbalance contribute to a hostile wound microenvironment that sustains chronic inflammation and impairs coordinated repair.

These processes collectively create a hostile wound microenvironment marked by immune imbalance, impaired vascular repair, and susceptibility to biofilm formation [15,16]. Dysbiosis within the wound microbiome may further exacerbate inflammatory signaling and disrupt host–microbe homeostasis, potentially sustaining delayed healing [16,31]. Experimental studies suggest that probiotic-derived metabolites and bacterial components can modulate inflammatory cytokine expression, enhance fibroblast migration, promote collagen deposition, and support angiogenesis [32,33,34,35].

The diabetic wound environment is also exceptionally vulnerable to pathogenic colonization. Bacterial growth is promoted by hyperglycemia and tissue ischemia, while bacterial clearance is reduced by immune dysfunction. Chronic wounds are typically colonised by polymicrobial biofilms composed mainly of *S. aureus*, *P. aeruginosa*, and anaerobic species [15]. Biofilms are highly resistant to antibiotics and host defense and produce toxin while maintaining inflammation [16]. Within this dysregulated environment, balance between commensal and pathogenic organisms is lost, thereby reinforcing infection, tissue destruction, and delayed healing. This microbial imbalance provides a strong biological rationale for exploring probiotics as a strategy to restore microbial equilibrium and support wound repair.

## 4. From Dysbiosis to Repair: The Therapeutic Logic of Probiotics

Healthy skin is an ecosystem composed of a diverse community of microorganisms, including bacteria, fungi, and viruses, that together form the skin microbiome [5]. In balance, these microbial residents play an important protective role: they compete with potential pathogens for nutrients and space, produce antimicrobial compounds, and help maintain an immune environment that supports tissue integrity. This symbiotic relationship between host and commensals contributes to skin resilience and normal wound repair.

In diabetic wounds, however, this equilibrium is disrupted. Persistent hyperglycaemia, impaired circulation and immune dysfunction create conditions that favour the growth of pathogenic species while reducing the abundance of protective commensals. Compared with contralateral healthy skin, the DFU microbiota shows less bacterial diversity with greater levels of opportunistic pathogens [36]. Studies have shown that chronic diabetic wounds often develop polymicrobial biofilms, with opportunistic pathogens such as *S. aureus* and *P. aeruginosa* dominating [15]. The synergistic cooperation between *S. aureus* and *P. aeruginosa* increases their tolerance to antibiotics, ability to form biofilms, and the secretion of virulence factors (hydrogen cyanide, exoenzyme S, exotoxin A, and pyocyanin for *P. aeruginosa*, and Panton–Valentine leukocidin and α hemolysin for *S. aureus*) that further damage tissue [37].

The loss of microbial diversity and the shift toward a pathogen-dominated ecosystem, commonly referred to as dysbiosis, is now recognised as a key barrier to wound healing, sustaining inflammation, delaying granulation tissue formation, and predisposing the wound to recurrent infection. Because this microbial imbalance plays such a central role in sustaining chronic inflammation and delayed healing, restoring a balanced wound microbiota has become an important therapeutic objective.

Within this complex environment of vascular impairment, neuropathy, immune dysfunction, microbial imbalance, and defective tissue repair, probiotics have therefore emerged as a promising therapeutic option by acting on both the wound microbiota and the host healing response. One of the most well-documented effects of probiotics is their ability to limit pathogen colonization by competing for adhesion sites and essential nutrients. Probiotics also interfere with quorum-sensing signaling through production of lactic acid, which lowers local pH, and other antimicrobial molecules such as hydrogen peroxide, reuterin, and bacteriocins. These substances are able to disrupt the most common chronic wound microbial pathogens or to inhibit their virulence [32,38].

Beyond their antimicrobial activity, probiotics play a critical role in disrupting biofilms, which represent one of the most important drivers of chronic wound persistence. An important antimicrobial mechanism involves regulation of antimicrobial peptide (AMPs) production by the host’s epithelial cells, adipocytes, and mast cells, which modulate skin integrity, reduce inflammation, and prevent adhesion and biofilm development [39].

*Lactiplantibacillus plantarum* (formerly *Lactobacillus plantarum*) is commonly used for the treatment of chronically infected wounds. In vitro studies have found that cell-free extracts of *L. plantarum* strains inhibit bacterial growth and bacterial biofilm formation of *P. aeruginosa*, methicillin-resistant *S. aureus* and hospital-derived skin pathogens [40]. *L. plantarum* extracts were also found to reduce the expression of population-sensing signals and soluble virulence factors of pathogenic bacteria required for biofilm formation. However, the exact mechanism of reducing bacterial biofilm formation was not well elaborated on in this study [40].

Furthermore, cell-free supernatants derived from *Lacticaseibacillus rhamnosus GG* were able to inhibit the formation of biofilms by *S. aureus* and *P. aeruginosa* by more than 95%, without causing resistance even after prolonged exposure [41,42].

Probiotics can also modulate the local immune response in wound healing by regulating cytokine production and influencing inflammatory cell recruitment [31]. They have been shown to increase anti-inflammatory cytokines such as IL-10 and TGF-β while reducing pro-inflammatory mediators like TNF-α, IL-6 and IL-1β as summarized in the selected studies [43,44]. In addition, probiotics can influence macrophage polarisation, encouraging a shift from the destructive M1 phenotype toward the reparative M2 phenotype, thereby promoting resolution of inflammation [45].

Another critical defect in diabetic wounds is impaired angiogenesis and keratinocyte migration, which delays tissue repair. Probiotic strains, including *Lacticaseibacillus casei* and *L. ramnosus GG (LGG)*, accelerate wound healing by releasing extracellular vesicles that promote the growth of new blood vessels (angiogenesis) and the formation of new skin (epithelialization). These vesicles contain factors like miR-21-5p, which they deliver to skin and endothelial cells, increasing their proliferation and migration to form new tissue [35,46]. In addition, *Bifidobacterium bifidum* has demonstrated potential in enhancing fibroblast activity and reducing bacterial burden in experimental models [33].

Together, these antimicrobial, antibiofilm, immunomodulatory and pro-angiogenic effects illustrate how probiotics can actively shift the chronic diabetic wound environment from persistent inflammation and infection toward improved microbial balance, controlled inflammation, neovascularisation, collagen deposition, and epithelial regeneration.

## 5. Evidence from Preclinical Studies

### 5.1. Animal Models of Topical Probiotics in Wound Healing

Experiments in animals provide the earliest evidence that probiotics can support the healing process. Most investigations have been performed in rodent models of diabetes or impaired healing, in which standardised excisional wounds are created and treated with topical probiotic formulations. The models allow researchers to quantify the direct effects of probiotics on wound closure, prevention of infection, and tissue repair under controlled conditions, including histological assessment of inflammation, angiogenesis, collagen deposition, and microbial burden. The key in vivo studies are summarized in Table 1.

### 5.2. Strains Investigated

A variety of probiotic strains have been tested in wound models.

Probiotic species most frequently investigated in diabetic wound models include *L. plantarum*, *L. rhamnosus*, *L. casei*, *Limosilactobacillus fermentum*, and *Lactobacillus delbrueckii*, among other lactic acid bacteria known for their antimicrobial and immunomodulatory properties [53]. *Lactiplantibacillus* species are Gram-positive, microaerophilic and non-sporulating microorganisms that modulate immune activity in the skin and mucosa and support cutaneous homeostasis [48].

Importantly, probiotic effects are strain-specific, and outcomes observed with one organism or formulation cannot be generalized across species or delivery systems [54].

Experimental studies have also explored combinations of strains, suggesting possible synergistic effects.

### 5.3. Outcomes Observed

Animal studies provide important insights into how probiotics may influence the impaired healing environment of diabetic wounds. In diabetic rat burn models, topical *L. plantarum* gels promoted faster wound closure, enhanced re-epithelialization, and helped reduce bacterial colonization [47].

Fermented soymilk extract–based formulations have also demonstrated anti-inflammatory activity, increased collagen deposition, and stimulated angiogenesis, pointing to both structural and vascular repair benefits [55].

Additional studies have shown that combinations of probiotic strains may provide broader benefits. For instance, *Lactobacillus bulgaricus* together with *L. plantarum* supported faster wound closure and modulated immune cell responses in diabetic rats [49]. A topical lysate of *Lactococcus chungangensis* applied to type 1 diabetic mice reduced wound size, lowered neutrophil (MPO) activity, and promoted cytokine and growth factor expression [50].

More advanced delivery systems are also being explored. A probiotic oleogel containing multiple strains (*Lactobacillus acidophilus*, *L. rhamnosus*, *L. fermentum*, and *L. casei*) improved granulation tissue formation, increased collagen density, stimulated angiogenesis, and disrupted biofilms in chronic diabetic ulcer models [51]. Most recently, a hydrogel combining *L. reuteri* with hydrogen-releasing nanoparticles demonstrated the ability to lower oxidative stress, scavenge excess glucose, and enhance angiogenesis, resulting in markedly faster closure of DFUs in mice [7].

In the nanocurcumin + viable *L. plantarum* dressing experiment, the combined treatment significantly sped up wound closure in mice compared to controls. The dressing reduced bacterial burden in the wound bed, lowered levels of inflammatory markers (TNF-α, MMP-9), and decreased oxidative damage (lipid peroxidation) [52]. It also boosted beneficial healing signals, including VEGF, TGF-β, and antioxidant enzymes like catalase and glutathione. Notably, the study found no evidence of probiotic translocation from the wound to internal organs, supporting topical safety in this experimental model [52].

However, these findings derive from immunocompetent animals with limited ischemic burden and short follow-up; therefore, their results should be interpreted cautiously and cannot be directly translated to high-risk human populations.

Together, these preclinical findings suggest that topical probiotics can positively influence multiple aspects of diabetic wound repair: they suppress infection and biofilms, reduce oxidative and inflammatory stress, stimulate fibroblast and collagen activity, and promote angiogenesis. While promising, these results remain preliminary and require validation in well-designed human trials before translation into clinical practice.

### 5.4. Limitations of Preclinical Evidence

Despite promising preclinical results, current animal models inadequately capture the full complexity of human diabetic wounds, especially neuropathy and vascular complications. This translational gap underscores the need for advanced models that better mimic human diabetic wound pathophysiology.

Another difficulty is the wide variety of probiotic strains and delivery systems used—from Lactobacillus in gels to experimental hydrogels—so that it is difficult to compare results or know which approaches are likely to work best.

Studies are also small, often with short follow-up periods, so we know little about long-term effects or optimal dosing.

Finally, safety remains insufficiently characterized, and the use of live microorganisms in open diabetic wounds raises understandable concerns about infection risk. Taken together, these considerations highlight that, while preclinical findings are encouraging, rigorous human studies are essential to confirm the true therapeutic value of topical probiotics in DFUs.

## 6. Evidence from Clinical Studies

### 6.1. Overview

So far, clinical research on topical probiotics in wound care is still limited, but interest is steadily growing, especially in the context of DFUs. Because these wounds are difficult to treat and often resistant to conventional therapies, they have naturally become the main focus of early probiotic trials [56,57,58].

Most of the published evidence comes from small pilot studies, single-center investigations, and isolated case reports, with no large randomized controlled trials currently available. Although these early studies offer useful preliminary insights into how microbiome-based therapies might influence wound healing in real clinical settings, they remain modest in scale. Their interpretation is further complicated by small patient numbers, differences in study populations, variation in probiotic strains and formulations, and a lack of standardized outcome measures. While modest in scale, these investigations provide early signs of how probiotics may work in real patients.

Moreover, several studies report limited methodological detail, including randomization procedures, baseline wound characteristics, and standardized definitions of healing. For example, the Taiwanese multicenter study reported high rates of ulcer closure but did not clearly describe patient allocation procedures, control interventions, or baseline comparability between groups, making interpretation of treatment effects more challenging and limiting direct comparison with established standard-of-care approaches [59].

Across these early reports, preliminary findings suggest possible trends toward faster wound closure and improved local wound conditions; however, these observations should be interpreted cautiously. Importantly, the absence of reported serious adverse events in small cohorts does not establish safety, particularly in high-risk DFU populations [56,57,58,59].

A range of delivery formats has been explored, including topical gels, creams, sprays, and probiotic-enriched dressings and although methods vary, the results point in the same general direction [57,58].

Across these early reports, probiotics have been linked with faster wound closure, better infection control, and healthier tissue repair. Just as importantly, they have been well tolerated, with no major safety concerns reported in DFU patients.

Taken together, the existing clinical literature should be viewed as hypothesis-generating rather than confirmatory. While early observations point to possible benefits, the current evidence base does not yet support definitive conclusions regarding either efficacy or safety. Larger, well-designed randomized controlled trials, using standardized protocols, clearly defined endpoints, and longer follow-up periods, are needed before topical probiotic therapies can be responsibly incorporated into routine DFU care.

### 6.2. Clinical Trials and Case Studies in DFUs

Table 2 brings together the clinical trials and case reports that have tested probiotics directly in DFUs.

### 6.3. Reported Outcomes

Overall, the available human evidence remains very limited and consists primarily of small pilot studies, a single-center case report, and one multicenter observational study, comprising fewer than 50 patients in total (Table 2). No large randomized controlled trials are currently available. Consequently, the existing literature should be regarded as hypothesis-generating rather than confirmatory.

One of the first pilot studies applied *L. plantarum* to chronically infected leg ulcers and found not only a reduction in bacterial load, but also signs of stronger immune activity and improved wound healing [57]. Building on this, a pilot trial in Argentina reported that weekly applications of *L. plantarum* after debridement were associated with faster healing of complicated DFUs, accompanied by neovascularization and a shift toward a more reparative immune profile helped complicated DFUs heal faster, with new blood vessel formation and a shift in immune cells toward a more reparative, healing profile [56].

Although the Taiwanese multicenter study reported that 83% of ulcers achieved closure within 16 weeks [59], it was conducted as a retrospective, uncontrolled evaluation involving 22 patients and without randomization or a comparator arm. While inclusion criteria and healing definitions were specified, the absence of a control group and the retrospective design limit causal interpretation of the observed outcomes. Moreover, only non-infected Wagner grade 1–2 ulcers were included, restricting generalizability to more complex DFU populations. In addition, the probiotic formulation used in this study was not clearly characterized at the strain level, which makes it more difficult to interpret the findings and compare them with other probiotic interventions. Taken together, these limitations make it challenging to draw firm conclusions regarding efficacy or to directly compare the results with standard care. These methodological constraints preclude robust comparison with standard care and prevent definitive conclusions regarding efficacy.

Individual case reports echo these findings. In one example, a woman with a chronic diabetic ulcer experienced complete healing after treatment with a multi-strain probiotic mixture (*L. acidophilus*, *L. plantarum*, *S. thermophilus*), which also eliminated persistent infections with *Proteus mirabilis* and *Klebsiella pneumoniae* [58].

Across these reports, preliminary findings suggest possible trends toward faster healing, healthier tissue repair, and better control of infection [57,58,59]. Just as importantly, no serious adverse events have been reported in the limited clinical studies available [57,58,59]; however, current sample sizes are insufficient to establish safety, particularly in high-risk DFU populations.

Taken together, current clinical findings suggest potential therapeutic promise but are constrained by small cohorts, heterogeneous probiotic formulations, variable delivery methods, and limited follow-up.

The absence of reported serious adverse events should not be interpreted as definitive evidence of safety.

Larger, well-designed randomized controlled trials with standardized outcome measures and long-term monitoring are required before conclusions regarding efficacy or safety can be drawn.

### 6.4. Limitations of Current Clinical Data

Although the early results are encouraging, the current clinical evidence has clear limitations. Most trials enrolled only a small number of patients, making it difficult to know if the positive results would hold in larger, more diverse populations. Follow-up times were generally short, meaning we do not yet know whether the benefits of probiotics last in the long term or if ulcers are more likely to recur after treatment. There is also considerable variation in the strains used (*L. plantarum* alone, multi-strain mixes, or soybean-based formulations), as well as in the delivery method (gels, dressings, or concentrates). Importantly, probiotic effects are highly strain-specific, and observed outcomes cannot be generalized across species or formulations, underscoring the need for precise strain characterization and head-to-head comparative studies. This heterogeneity makes direct comparison between studies challenging and prevents us from identifying the “best” probiotic approach. On top of that, most reports come from single-centre or pilot studies, with only a handful of randomised controlled trials to date. Together, these gaps underline the need for larger, multicentre studies with stronger designs before probiotics can be confidently recommended as part of standard DFU care.

### 6.5. Safety Considerations in Microbiome-Based Topical Therapies

The topical application of live microorganisms to chronic diabetic wounds raises important safety considerations that should be addressed before clinical translation. Patients with DFUs commonly present with peripheral arterial disease and tissue hypoxia, which can profoundly shape the wound microenvironment and the host response to microbial exposure. Peripheral arterial disease is reported in a substantial proportion of people with DFUs, reaching up to ~50% in some settings and contributes significantly to impaired perfusion and chronic wound pathology [60]. In severely ischemic wounds characterized by marked tissue hypoxia, it also remains unclear whether microaerophilic probiotic strains can maintain viability or whether altered oxygen tension could influence their behavior in ways not yet fully understood.

Chronic inflammation and immune dysfunction impair the healing process of DFUs, leading to persistent infections and tissue damage. In this context, impaired immune responses reduce bacterial clearance and increase susceptibility to infection, further contributing to wound chronicity [14].

Importantly, the current human evidence base for topical live probiotics in DFUs remains small and heterogeneous, and is underpowered to detect uncommon but clinically meaningful adverse events such as bacteremia or sepsis [57,58,59]. For this reason, the absence of reported serious adverse events in early studies should be interpreted cautiously rather than considered confirmatory evidence of safety.

Although preclinical studies have suggested limited systemic spread after topical application, these findings are largely derived from immunocompetent animal models and cannot be directly extrapolated to neuro-ischemic DFUs in humans, where multifactorial tissue pathology is present [56]. As such, apparent short-term safety in experimental models does not necessarily predict outcomes in clinically complex DFU populations.

In addition, chronic kidney disease frequently co-exists with diabetes and DFUs, further increasing clinical complexity and worsening outcomes [61]. Moreover, many patients with DFUs are treated concurrently with systemic antibiotics, which can substantially alter wound microbiota and immune responses, potentially influencing both probiotic activity and safety outcomes [2]. Additionally, interactions between administered strains and established polymicrobial biofilms in chronic DFUs remain insufficiently characterized, representing another key safety and efficacy uncertainty [15,16].

In this context, postbiotic strategies—including bacterial lysates, cell-free supernatants, and microbial metabolites—have attracted increasing interest as potentially safer and more controllable alternatives to live microorganisms. These approaches aim to preserve antimicrobial and immunomodulatory effects while reducing the theoretical risks associated with applying viable bacteria to already compromised tissue [31,32,34,62].

For high-risk patients in particular, postbiotic-based formulations may therefore represent a more cautious and clinically acceptable strategy until robust safety data for live probiotic therapies become available.

Taken together, these considerations highlight the need for carefully designed clinical studies that include patient risk stratification, standardized safety endpoints, and longer follow-up periods, as well as clearer guidance on how microbiome-based therapies could be integrated into established wound care pathways.

## 7. Innovations in Probiotic Delivery

A major challenge in translating probiotic-based strategies into routine diabetic wound care lies not only in proving their effectiveness, but also in ensuring safe and effective delivery to the wound site. Traditional gels and ointments have shown promise, but researchers are now exploring more innovative approaches designed to enhance stability, prolong activity, and maximize therapeutic benefit [7,34,52].

One approach involves bioengineered dressings and biomaterials incorporating live probiotic strains into wound coverings, functioning both as protective barriers and sustained-release platforms. Hydrogels and electrospun nanofiber scaffolds embedding *L. plantarum* and *L. reuteri* have demonstrated accelerated wound closure, improved infection control, and enhanced angiogenesis in preclinical models [31]. Nevertheless, the use of viable microorganisms raises important safety considerations, particularly in patients with ischemia, immunosuppression, or multiple comorbidities, where bacterial translocation or uncontrolled colonization cannot be fully excluded.

In parallel, growing interest has shifted toward postbiotics and probiotic-derived metabolites. Instead of applying live organisms, these approaches use bacterial components or secreted products—such as short-chain fatty acids, antimicrobial peptides, or cell-free supernatants—that can mimic the beneficial effects of probiotics without the risks associated with viable bacteria [32,33,34,35,36,37,38,39,40,41,42,43,44,45,46,47,48,49,50,51,52,53,54,55,56,57,58,59,60,61,62,63]. Experimental evidence indicates that postbiotic preparations from *Lactococcus* and *Lactiplantibacillus* species can attenuate inflammation, stimulate fibroblast activity, and support tissue repair in diabetic wound models [31].

In this context, postbiotic-based formulations may offer a more controlled and potentially safer strategy, particularly for high-risk patient populations, until more robust safety data for live probiotic therapies become available.

Additional innovation includes symbiotic approaches combining probiotics with prebiotics, which have demonstrated synergistic antimicrobial and immune-enhancing effects, as well as artificial intelligence-driven personalized medicine strategies using microbiome profiling to tailor interventions to individual wound environments [31]. Furthermore, multimodal therapies pairing probiotics or postbiotics with antibiotics, growth factors, or stem cell-based treatments are being explored to enhance healing outcomes and overcome resistant infections [8,31].

Although these approaches remain largely experimental, they reflect a broader shift toward precision-oriented and safety-conscious microbiome-based wound care. By improving stability and harnessing synergistic strategies, future approaches may help probiotic-derived therapies achieve their full therapeutic potential in diabetic wound care.

## 8. Discussion

### Challenges and Future Research Directions

DFUs represent one of the most challenging and costly complications of diabetes, with high rates of delayed healing and recurrence despite advances in current therapeutic strategies [1,2]. Within this therapeutic gap, topical probiotics have emerged as a promising, though still experimental, approach.

Preclinical animal studies and early data suggest that topical probiotics may influence wound healing by modulating inflammation, reducing pathogenic bacterial burden, and enhancing tissue repair processes such as collagen deposition and epithelialization; however, evidence in diabetic wound models remains limited [39,53].

Early clinical studies, though modest in scale, broadly support these experimental observations. Topical application of *L. plantarum* and other probiotic formulations in DFUs have been linked to faster wound closure, improved tissue characteristics and better control of bacterial burden, with no serious adverse events reported in small cohorts [56,57,58]. While these outcomes are encouraging, they must be interpreted with caution. Trials so far have involved small numbers of patients, varied strains and delivery methods, and limited follow-up, leaving important questions about durability, reproducibility, and optimal formulation unanswered [8,39].

Collectively, these reports comprise small pilot studies and observational clinical evaluations in which topical probiotic formulations were applied as adjuncts to standard wound care. Reported outcomes typically included time to closure, proportion of ulcers healed, changes in local bacterial burden, and qualitative improvements in granulation tissue and wound appearance [57,58,59]. Although several studies reported faster healing and fewer signs of local infection, these findings should be interpreted cautiously. Differences in probiotic formulations and application schedules, short follow-up periods, and inconsistent reporting of baseline ulcer severity and concurrent standard care all limit direct comparison across studies. In addition, most investigations lacked robust control groups, making it difficult to draw firm causal conclusions.

Taken together, the available clinical data should be viewed as preliminary and hypothesis-generating rather than confirmatory.

Looking ahead, the field is moving into more innovative territory. Bioengineered dressings, postbiotic formulations, and synergistic strategies that combine probiotics with antibiotics, growth factors, or stem cells are being explored to improve stability and potency [31,32,34]. These developments highlight the versatility of probiotics and their derivatives as future therapeutic tools.

To establish topical probiotics as a mainstream therapeutic approach for diabetic wounds, several pivotal gaps must be addressed. First, safety concerns related to applying live microorganisms in chronic wounds, particularly among immunocompromised patients, require rigorous investigation and clear regulatory guidelines [8,31].

Importantly, probiotic- and postbiotic-based interventions should be regarded as adjunctive therapies rather than replacements for established evidence-based DFU management. Optimal outcomes continue to depend on comprehensive standard care, including regular sharp debridement, appropriate offloading to reduce mechanical stress, and assessment of vascular status with revascularization when indicated [1,2]. In clinical practice, topical probiotic-derived applications would be expected to follow adequate wound bed preparation and be integrated alongside conventional measures such as moisture-balanced dressings and infection surveillance. Potential interactions with commonly used topical antiseptics, including iodine-, silver-, and polyhexamethylene biguanide-based agents, also warrant consideration, as these may impair microbial activity. Likewise, systemic antibiotics remain essential for clinically infected ulcers, and probiotic or postbiotic approaches should be viewed as complementary rather than substitutive. Future studies should explicitly report concurrent standard-of-care practices to better define real-world applicability.

Furthermore, the high heterogeneity of probiotic strains and delivery systems necessitates comparative and synergy-focused research to identify the most effective therapeutic agents and delivery formulations [39,55]. Personalized medicine approaches, leveraging comprehensive wound microbiome and immune profiling, offer an opportunity to tailor probiotic interventions to the individual patient’s wound environment for improved outcomes [8].

Innovative technologies such as nanoencapsulation, stimulus-responsive hydrogels, and integration of biosensors offer exciting prospects for more intelligent and targeted probiotic delivery [7,52]. The clinical translation of these advanced modalities will require multidisciplinary efforts and well-designed clinical trials to optimise efficacy, safety, and patient acceptability [8].

Addressing these challenges may help advance topical probiotics from experimental adjuncts toward more evidence-based, precision-oriented therapies.

## 9. Conclusions

In summary, topical probiotics represent an important step toward more holistic, microbiome-informed management of diabetic wounds. While they cannot yet be considered part of standard care, preliminary signals from early studies, their preliminary tolerability in limited studies, and growing technological innovations suggest areas for further investigation

However, current evidence remains insufficient to establish definitive efficacy or safety, particularly in high-risk DFU populations. With rigorous, multicenter clinical trials and thoughtful integration into existing wound care protocols, probiotics could move from promising adjuncts to established partners in the fight against DFUs.

## Figures and Tables

**Table 1:** Summary of in vivo studies investigating topical probiotics in diabetic foot healing, focused on diabetic wounds. The table highlights study type, author and year, wound model, probiotic formulation, experimental model/patients, and reported in vitro activities. ↑ indicates increase; ↓ indicates decrease.

Study Type	Author/Year	Wound Type/Model	Probiotic/Formulation	Application	Key Outcomes
In vivo (Rats, diabetic)	Salaran 2019 [47]	Burn wounds in diabetic rats	*L. plantarum* gel	Topical	Faster re-epithelialisation, infection control
In vivo (Diabetic rats)	Ong et al., 2020 [48]	Excision/infected wound model	*L. plantarum* USM8613	Topical	Faster wound closure, reduced *S. aureus*, improved healing
In vivo (Diabetic rats)	Mohtashami 2021 [49]	Diabetes-induced wounds	*L. bulgaricus*, *L. plantarum*	Topical, 14 d	Faster closure, immune modulation
In vivo (Mice, T1D)	Nam 2021 [50]	Type 1 diabetes-induced wounds	*Lactococcus chungangensis* CAU 1447 lysate	Wound dressing, 7 d	↓ wound size, ↓ MPO, ↑ cytokines & growth factors
In vivo (Rats, diabetic ulcer)	Karimi 2024 [51]	Diabetic ulcer	Oleogel with *L. acidophilus*, *L. rhamnosus*, *L. fermentum*, *L. casei*	Topical oleogel	↑ granulation, collagen, angiogenesis, antibiofilm
In vivo (Mice)	Sandu et al. 2023 [52]	Excision wound model	*L. plantarum* in nanocurcumin-loaded wound dressing	Topical dressing	Faster closure, ↓ bacterial load, ↓ TNF-α/MMP-9, ↑ VEGF, TGF-β, antioxidant enzymes, no systemic spread
In vivo (Mice, DFU)	Wang 2025 [7]	Diabetic foot ulcer model	*L. reuteri* hydrogel + H_2_ nanoparticles	Topical hydrogel	↓ ROS, glucose scavenging, faster closure

**Table 2:** Overview of published clinical studies investigating topical probiotic therapies in diabetic or chronic wound healing. Current evidence consists of one small randomized controlled trial, retrospective observational data, pilot studies, and isolated case reports. ↑ indicates increase; ↓ indicates decrease.

Study	Study Design	Population	Probiotic/Formulation	Application	Key Outcomes	Limitations
Argentina 2022 [56]	Randomized controlled trial (n = 22)	Patients with complicated diabetic foot ulcers (DFUs) post-debridement (SuDe n = 12 vs. SuDe + *L. plantarum* n = 10)	*L. plantarum*	Topical, weekly for 12 weeks	Greater wound area reduction (73.5% vs. 45.8%); higher closure rates; increased fibroplasia and angiogenesis; M2 macrophage polarization; reduced bacterial burden	Small sample size; short follow-up; single center
Yang et al., 2023 (Taiwan) [59]	Retrospective multicenter observational study	Non-infected DFUs (n = 22; Wagner grade 1–2)	Probiotic soybean-based concentrate	Topical, twice daily	83% achieved complete closure by 16 weeks; reduced healing time (retrospective, uncontrolled)	Retrospective design; no control group; limited generalizability
Peral et al., 2010 [57]	Pilot clinical study	14 diabetic + 20 non-diabetic patients with chronic infected leg ulcers	*L. plantarum*	Topical	↓ bacterial load, ↑ immune cells, faster healing, improved inflammatory profile	Pilot study; no control group
Venosi et al., 2019 [58]	Case report	83-year-old woman with diabetes	*L. acidophilus* NCIMB 43030, *L. plantarum* NCIMB 43029, *S. thermophilus* NCIMB 30438	Topical, 24 days	Complete wound healing, eradication of P. mirabilis and K. pneumoniae	Single patient

## Data Availability

No new data were created or analyzed in this study. Data sharing is not applicable to this article.

## References

[1] Armstrong D.G., Boulton A.J.M., Bus S.A. (2017). Diabetic Foot Ulcers and Their Recurrence. N. Engl. J. Med..

[2] Schaper N.C., van Netten J.J., Apelqvist J., Bus S.A., Fitridge R., Game F., Monteiro-Soares M., Senneville E., Board I.E. (2024). Practical guidelines on the prevention and management of diabetes-related foot disease (IWGDF 2023 update). Diabetes Metab. Res. Rev..

[3] Feng J., Lai S., Tang D. (2026). Mechanisms and Interventions of Diabetic Wound Healing. Curr. Diabetes Rev..

[4] Chinemerem Nwobodo D., Ugwu M.C., Oliseloke Anie C., Al-Ouqaili M.T.S., Chinedu Ikem J., Victor Chigozie U., Saki M. (2022). Antibiotic resistance: The challenges and some emerging strategies for tackling a global menace. J. Clin. Lab. Anal..

[5] Grice E.A., Segre J.A. (2011). The skin microbiome. Nat. Rev. Microbiol..

[6] Falanga V. (2005). Wound healing and its impairment in the diabetic foot. Lancet.

[7] Wang Y., Shi L., Lu J., Wang F., Zhou Z., Wang Y., Du X., Qin D., Chen F., Shao D. (2025). Probiotic active gel promotes diabetic wound healing through continuous local glucose consumption and antioxidant. J. Nanobiotechnol..

[8] Hussain A., Mojgani N., Shah S.M.A., Kousar N., Ali S.A. (2025). The emerging role of probiotics in the management and treatment of diabetic foot ulcer: A comprehensive review. AIMS Microbiol..

[9] Zheng J., Wittouck S., Salvetti E., Franz C.M.A.P., Harris H.M.B., Mattarelli P., O’Toole P.W., Pot B., Vandamme P., Walter J. (2020). A taxonomic note on the genus *Lactobacillus*: Description of 23 novel genera, emended description of the genus *Lactobacillus* Beijerinck 1901, and union of *Lactobacillaceae* and *Leuconostocaceae*. Int. J. Syst. Evol. Microbiol..

[10] Cade W.T. (2008). Diabetes-related microvascular and macrovascular diseases in the physical therapy setting. Phys. Ther..

[11] Szabo C. (2009). Role of nitrosative stress in the pathogenesis of diabetic vascular dysfunction. Br. J. Pharmacol..

[12] Rask-Madsen C., King G.L. (2013). Vascular complications of diabetes: Mechanisms of injury and protective factors. Cell Metab..

[13] Khamaisi M., Katagiri S., Keenan H., Park K., Maeda Y., Li Q., Qi W., Thomou T., Eschuk D., Tellechea A. (2016). PKCdelta inhibition normalizes the wound-healing capacity of diabetic human fibroblasts. J. Clin. Investig..

[14] Dawi J., Tumanyan K., Tomas K., Misakyan Y., Gargaloyan A., Gonzalez E., Hammi M., Tomas S., Venketaraman V. (2025). Diabetic Foot Ulcers: Pathophysiology, Immune Dysregulation, and Emerging Therapeutic Strategies. Biomedicines.

[15] Pouget C., Dunyach-Remy C., Pantel A., Schuldiner S., Sotto A., Lavigne J.P. (2020). Biofilms in Diabetic Foot Ulcers: Significance and Clinical Relevance. Microorganisms.

[16] Afonso A.C., Oliveira D., Saavedra M.J., Borges A., Simoes M. (2021). Biofilms in Diabetic Foot Ulcers: Impact, Risk Factors and Control Strategies. Int. J. Mol. Sci..

[17] Deng L., Du C., Song P., Chen T., Rui S., Armstrong D.G., Deng W. (2021). The Role of Oxidative Stress and Antioxidants in Diabetic Wound Healing. Oxidative Med. Cell. Longev..

[18] Khaodhiar L., Dinh T., Schomacker K.T., Panasyuk S.V., Freeman J.E., Lew R., Vo T., Panasyuk A.A., Lima C., Giurini J.M. (2007). The use of medical hyperspectral technology to evaluate microcirculatory changes in diabetic foot ulcers and to predict clinical outcomes. Diabetes Care.

[19] Krishnan S.T., Rayman G. (2004). The LDIflare: A novel test of C-fiber function demonstrates early neuropathy in type 2 diabetes. Diabetes Care.

[20] Ceradini D.J., Kulkarni A.R., Callaghan M.J., Tepper O.M., Bastidas N., Kleinman M.E., Capla J.M., Galiano R.D., Levine J.P., Gurtner G.C. (2004). Progenitor cell trafficking is regulated by hypoxic gradients through HIF-1 induction of SDF-1. Nat. Med..

[21] Guo S., Dipietro L.A. (2010). Factors affecting wound healing. J. Dent. Res..

[22] Rodrigues M., Kosaric N., Bonham C.A., Gurtner G.C. (2019). Wound Healing: A Cellular Perspective. Physiol. Rev..

[23] Rodrigues M., Wong V.W., Rennert R.C., Davis C.R., Longaker M.T., Gurtner G.C. (2015). Progenitor cell dysfunctions underlie some diabetic complications. Am. J. Pathol..

[24] Wetzler C., Kampfer H., Stallmeyer B., Pfeilschifter J., Frank S. (2000). Large and sustained induction of chemokines during impaired wound healing in the genetically diabetic mouse: Prolonged persistence of neutrophils and macrophages during the late phase of repair. J. Investig. Dermatol..

[25] Khanna S., Biswas S., Shang Y., Collard E., Azad A., Kauh C., Bhasker V., Gordillo G.M., Sen C.K., Roy S. (2010). Macrophage dysfunction impairs resolution of inflammation in the wounds of diabetic mice. PLoS ONE.

[26] Wolf S.J., Melvin W.J., Gallagher K. (2021). Macrophage-mediated inflammation in diabetic wound repair. Semin. Cell Dev. Biol..

[27] Wilkinson H.N., Clowes C., Banyard K.L., Matteuci P., Mace K.A., Hardman M.J. (2019). Elevated Local Senescence in Diabetic Wound Healing Is Linked to Pathological Repair via CXCR2. J. Investig. Dermatol..

[28] Liu Z.J., Velazquez O.C. (2008). Hyperoxia, endothelial progenitor cell mobilization, and diabetic wound healing. Antioxid. Redox Signal..

[29] Yamagishi S., Imaizumi T. (2005). Diabetic vascular complications: Pathophysiology, biochemical basis and potential therapeutic strategy. Curr. Pharm. Des..

[30] Guo J., Cao Y., Wu Q.Y., Zhou Y.M., Cao Y.H., Cen L.S. (2024). Implications of pH and Ionic Environment in Chronic Diabetic Wounds: An Overlooked Perspective. Clin. Cosmet. Investig. Dermatol..

[31] Machado P., Ribeiro F.N., Giublin F.C.W., Mieres N.G., Tonin F.S., Pontarolo R., Sari M.H.M., Lazo R.E.L., Ferreira L.M. (2025). Next-Generation Wound Care: A Scoping Review on Probiotic, Prebiotic, Synbiotic, and Postbiotic Cutaneous Formulations. Pharmaceuticals.

[32] Pique N., Berlanga M., Minana-Galbis D. (2019). Health Benefits of Heat-Killed (Tyndallized) Probiotics: An Overview. Int. J. Mol. Sci..

[33] Bazjoo A., Jafari P., Marjani A., Akbari N. (2022). Effect of Cell-Free Supernatant of Bifidobacterium bifidum Combined with Chitosan Biodegradable Film on Full Thickness Wound Healing in Rats. Physiol. Pharmacol..

[34] Tsai W.H., Chou C.H., Huang T.Y., Wang H.L., Chien P.J., Chang W.W., Lee H.T. (2021). Heat-Killed Lactobacilli Preparations Promote Healing in the Experimental Cutaneous Wounds. Cells.

[35] Wang J., Li X., Zhao X., Yuan S., Dou H., Cheng T., Huang T., Lv Z., Tu Y., Shi Y. (2024). Lactobacillus rhamnosus GG-derived extracellular vesicles promote wound healing via miR-21-5p-mediated re-epithelization and angiogenesis. J. Nanobiotechnol..

[36] Gontcharova V., Youn E., Sun Y., Wolcott R.D., Dowd S.E. (2010). A comparison of bacterial composition in diabetic ulcers and contralateral intact skin. Open Microbiol. J..

[37] Hotterbeekx A., Kumar-Singh S., Goossens H., Malhotra-Kumar S. (2017). In vivo and In vitro Interactions between *Pseudomonas aeruginosa* and *Staphylococcus* spp.. Front. Cell. Infect. Microbiol..

[38] Maldonado Galdeano C., Cazorla S.I., Lemme Dumit J.M., Velez E., Perdigon G. (2019). Beneficial Effects of Probiotic Consumption on the Immune System. Ann. Nutr. Metab..

[39] Knackstedt R., Knackstedt T., Gatherwright J. (2020). The role of topical probiotics on wound healing: A review of animal and human studies. Int. Wound J..

[40] Onbas T., Osmanagaoglu O., Kiran F. (2019). Potential Properties of *Lactobacillus plantarum* F-10 as a Bio-control Strategy for Wound Infections. Probiotics Antimicrob. Proteins.

[41] Meroni G., Panelli S., Zuccotti G., Bandi C., Drago L., Pistone D. (2021). Probiotics as therapeutic tools against pathogenic biofilms: Have we found the perfect weapon?. Microbiol. Res..

[42] Pompilio A., Kaya E., Lupetti V., Catelli E., Bianchi M., Maisetta G., Esin S., Di Bonaventura G., Batoni G. (2024). Cell-free supernatants from *Lactobacillus* strains exert antibacterial, antibiofilm, and antivirulence activity against *Pseudomonas aeruginosa* from cystic fibrosis patients. Microbes Infect..

[43] Mei L., Zhang D., Shao H., Hao Y., Zhang T., Zheng W., Ji Y., Ling P., Lu Y., Zhou Q. (2022). Injectable and Self-Healing Probiotics-Loaded Hydrogel for Promoting Superbacteria-Infected Wound Healing. ACS Appl. Mater. Interfaces.

[44] Dubey A.K., Sharma M., Parul, Raut S., Gupta P., Khatri N. (2023). Healing wounds, defeating biofilms: *Lactiplantibacillus plantarum* in tackling MRSA infections. Front. Microbiol..

[45] Zhou C., Zou Y., Xu R., Han X., Xiang Z., Guo H., Li X., Liang J., Zhang X., Fan Y. (2023). Metal-phenolic self-assembly shielded probiotics in hydrogel reinforced wound healing with antibiotic treatment. Mater. Horiz..

[46] De Rudder C., Garcia-Timermans C., De Boeck I., Lebeer S., Van de Wiele T., Calatayud Arroyo M. (2020). *Lacticaseibacillus casei* AMBR2 modulates the epithelial barrier function and immune response in a donor-derived nasal microbiota manner. Sci. Rep..

[47] Salaran M., Oryan A., Nikahval B., Kamali A., Ghaemi M., Abbasi-Teshnizi F., Azizzadeh M. (2019). Topical Application of Lactobacillus Plantarum on Burn Wound Healing in Diabetic Rats. Iran. J. Vet. Surg..

[48] Ong J.S., Taylor T.D., Yong C.C., Khoo B.Y., Sasidharan S., Choi S.B., Ohno H., Liong M.T. (2020). *Lactobacillus plantarum* USM8613 Aids in Wound Healing and Suppresses Staphylococcus aureus Infection at Wound Sites. Probiotics Antimicrob. Proteins.

[49] Mohtashami M., Mohamadi M., Azimi-Nezhad M., Saeidi J., Nia F.F., Ghasemi A. (2021). *Lactobacillus bulgaricus* and *Lactobacillus plantarum* improve diabetic wound healing through modulating inflammatory factors. Biotechnol. Appl. Biochem..

[50] Nam Y., Kim J., Baek J., Kim W. (2021). Improvement of Cutaneous Wound Healing via Topical Application of Heat-Killed Lactococcus chungangensis CAU 1447 on Diabetic Mice. Nutrients.

[51] Karimi F., Montazeri-Najafabady N., Mohammadi F., Azadi A., Koohpeyma F., Gholami A. (2024). A potential therapeutic strategy of an innovative probiotic formulation toward topical treatment of diabetic ulcer: An in vivo study. Nutr. Diabetes.

[52] Kaur Sandhu S., Raut J., Kumar S., Singh M., Ahmed B., Singh J., Rana V., Rishi P., Ganesh N., Dua K. (2023). Nanocurcumin and viable *Lactobacillus plantarum* based sponge dressing for skin wound healing. Int. J. Pharm..

[53] Gudadappanavar A.M., Hombal P.R., Timashetti S.S., Javali S.B. (2017). Influence of *Lactobacillus acidophilus* and *Lactobacillus plantarum* on wound healing in male Wistar rats—An experimental study. Int. J. Appl. Basic Med. Res..

[54] Hill C., Guarner F., Reid G., Gibson G.R., Merenstein D.J., Pot B., Morelli L., Canani R.B., Flint H.J., Salminen S. (2014). Expert consensus document. The International Scientific Association for Probiotics and Prebiotics consensus statement on the scope and appropriate use of the term probiotic. Nat. Rev. Gastroenterol. Hepatol..

[55] Lukic J., Chen V., Strahinic I., Begovic J., Lev-Tov H., Davis S.C., Tomic-Canic M., Pastar I. (2017). Probiotics or pro-healers: The role of beneficial bacteria in tissue repair. Wound Repair Regen..

[56] Arganaraz Aybar J.N., Ortiz Mayor S., Olea L., Garcia J.J., Nisoria S., Kolling Y., Melian C., Rachid M., Torres Dimani R., Werenitzky C. (2022). Topical Administration of *Lactiplantibacillus plantarum* Accelerates the Healing of Chronic Diabetic Foot Ulcers through Modifications of Infection, Angiogenesis, Macrophage Phenotype and Neutrophil Response. Microorganisms.

[57] Peral M.C., Rachid M.M., Gobbato N.M., Huaman Martinez M.A., Valdez J.C. (2010). Interleukin-8 production by polymorphonuclear leukocytes from patients with chronic infected leg ulcers treated with *Lactobacillus plantarum*. Clin. Microbiol. Infect..

[58] Venosi S., Ceccarelli G., de Angelis M., Laghi L., Bianchi L., Martinelli O., Maruca D., Cavallari E.N., Toscanella F., Vassalini P. (2019). Infected chronic ischemic wound topically treated with a multi-strain probiotic formulation: A novel tailored treatment strategy. J. Transl. Med..

[59] Yang C.C., Wu M.S., Hsu H. (2023). Management of diabetic foot ulcers using topical probiotics in a soybean-based concentrate: A multicentre study. J. Wound Care.

[60] Meloni M., Vas P.R.J. (2025). Peripheral Arterial Disease in Diabetic Foot: One Disease with Multiple Patterns. J. Clin. Med..

[61] Bonnet J.B., Sultan A. (2022). Narrative Review of the Relationship Between CKD and Diabetic Foot Ulcer. Kidney Int. Rep..

[62] Jamaran S., Jafari P., Marjani A., Akbari N., Feizabad M.M. (2021). Novel Wound Dressing Based on Postbiotic/Chitosan Film Accelerates Cutaneous Wound Healing. Jundishapur J. Microbiol..

[63] Żółkiewicz J., Marzec A., Ruszczyński M., Feleszko W. (2020). Postbiotics-A Step Beyond Pre- and Probiotics. Nutrients.

